# Multiple Access Control for Cognitive Radio-Based IEEE 802.11ah Networks

**DOI:** 10.3390/s18072043

**Published:** 2018-06-26

**Authors:** Muhammad Shafiq, Maqbool Ahmad, Azeem Irshad, Moneeb Gohar, Muhammad Usman, Muhammad Khalil Afzal, Jin-Ghoo Choi, Heejung Yu

**Affiliations:** 1 Department of Information Technology, University of Gujrat, Gujrat 50700, Pakistan; shafiq.pu@gmail.com; 2 Department of Digital Convergence Business, Yeungnam University, Gyeongsan 38541, Korea; maqbool.pu@gmail.com; 3 Department of Computer Science & Software Engineering, International Islamic University, Islamabad 44000, Pakistan; irshadazeem2@gmail.com; 4 Department of Computer Science, Bahria University, Islamabad 44000, Pakistan; moneebgohar@gmail.com; 5 Department of Computer Science, Quaid-i-Azam University, Islamabad 44000, Pakistan; musman@qau.edu.pk; 6 Department of Computer Science, COMSATS Institute of Information Technology, Wah Cantt 47040, Pakistan; khalilafzal@ciitwah.edu.pk; 7 Department of Information and Communication Engineering, Yeungnam University, Gyeongsan 38541, Korea; heejung@yu.ac.kr

**Keywords:** carrier sensing, CSMA/CA, dynamic spectrum access, spectrum sensing, IoT

## Abstract

The proliferation of Internet-of-Things (IoT) technology and its reliance on the license-free Industrial, Scientific, and Medical (ISM) bands have rendered radio spectrum scarce. The IoT can nevertheless obtain great advantage from Cognitive Radio (CR) technology for efficient use of a spectrum, to be implemented in IEEE 802.11af-based primary networks. However, such networks require a geolocation database and a centralized architecture to communicate white space information on channels. On the other hand, in spectrum sensing, CR presents various challenges such as the Hidden Primary Terminal (HPT) problem. To this end, we focus on the most recently released standard, i.e., IEEE 802.11ah, in which IoT stations can first be classified into multiple groups to reduce collisions and then they can periodically access the channel. Therein, both services are similarly supported by a centralized server that requires signaling overhead to control the groups of stations. In addition, more regroupings are required over time due to the frequent variations in the number of participating stations, which leads to more overhead. In this paper, we propose a new Multiple Access Control (MAC) protocol for CR-based IEEE 802.11ah systems, called Restricted Access with Collision and Interference Resolution (RACIR). We introduce a decentralized group split algorithm that distributes the participating stations into multiple groups based on a probabilistic estimation in order to resolve collisions. Furthermore, we propose a decentralized channel access procedure that avoids the HPT problem and resolves interference with the incumbent receiver. We analyze the performance of our proposed MAC protocol in terms of normalized throughput, packet delay and energy consumption with the Markov model and analytic expressions. The results are quite promising, which makes the RACIR protocol a strong candidate for the CR-based IoT environment.

## 1. Introduction

Radio spectrum is undoubtedly a vital resource that needs to be managed effectively. Spectrum is a kind of natural resource like water, gas, land, and minerals; but unlike them, spectrum is reusable over spatial and temporal dimensions. Usually, it is divided into discrete bands ranging between 3 kHz and 300 GHz, having multiple licensed and unlicensed bands. Within each band, various frequencies are distributed into small sets of non-overlapping frequencies (or channels) to prevent interference. Radio frequencies are traditionally assigned to groups of similar services for long-term periods by static spectrum allocation [1]. In this method, each channel is exclusively assigned to a single service provider, which often leads to a huge waste of spectrum [2,3,4].

Currently, the Internet-of-Things (IoT) has become an increasingly growing technology due to colossal evolution and integration of tiny objects like Radio Frequency ID (RFID) tags, sensors, actuators, etc., with the Internet. The IoT is now taking hold in almost every discipline of life in order to liberate humans from old and dumb devices with new low-power and low-cost smart objects that can operate independently. The development of these smart objects has enabled IoT technology to offer a wide range of useful applications such as smart homes, smart agriculture, smart grids, smart cars, connected health, and so on [5]. Under these applications, a lot of battery-powered smart objects essentially require Internet connectivity anywhere, anytime for anything. Unfortunately, most of the IoT-enabling technologies such as ZigBee, WiFi, 6LoWPAN, Bluetooth Low Energy (LE), etc., rely on the license-free Industrial, Scientific, and Medical (ISM) bands for spectrum. The increasing demand for spectrum requires makeshift changes in the traditional static spectrum allocation policy to protect against the ISM bands becoming congested.

**Figure 1:**
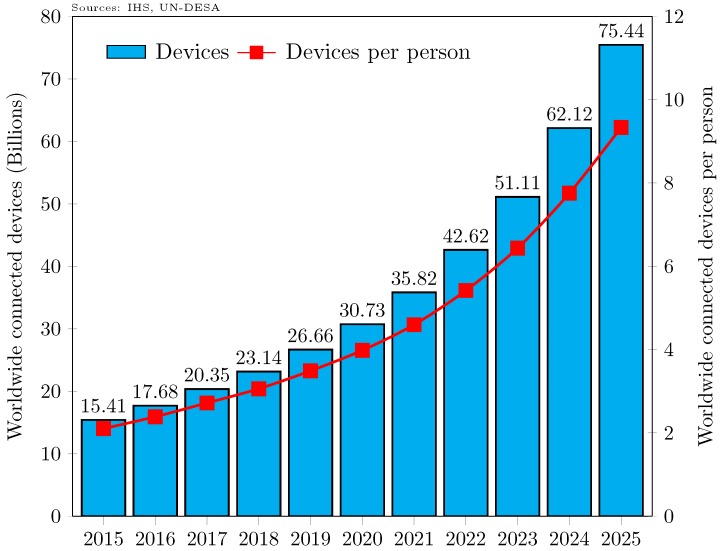
Internet-connected devices from 2015 to 2025.

Limited by static allocations and technological inventions like 3G and 4G telecom services, spectrum scarcity is considered one of the major issues for both the industry and academia. One recent report anticipates that 2.7% of physical objects will have smart devices attached to them by 2020, whereas the portion was only 0.6% in 2012 [6]. Furthermore, based on the United Nations world population prospects [7] and information from another recent report [8], we calculate that, in 2025, each person will have at least nine things with Internet-connected smart devices on average, as shown in Figure 1. Parts of the spectrum necessarily accommodate new inventions and emerging demand from the Internet, so that technological innovation could gear up with better utilization of spectrum, maximizing net gains and social benefits. Thus, the rapid proliferation of the IoT necessitates the development of new access paradigms, enabling protocols, and more spectrum.

In much of the research, we find proposals to use the Cognitive Radio (CR) system [9,10,11,12], in order to resolve the spectrum shortage issue for IoT-enabled networks. In CR technology, Secondary Users (SUs) can exploit the licensed channels whenever the legitimate Primary Users (PUs) are inactive. Meanwhile, SUs are obligated to immediately vacate the channel once PUs become active. Otherwise, PUs can suffer harmful interference from the SUs’ transmissions. However, the temporal and spatial appearance of PUs and imperfect sensing results make it really difficult to avoid interfering with PUs. Furthermore, CR networks, especially with a decentralized architecture, are prone to the hidden primary terminal problem [13,14,15], which can also interfere with PUs.

In Chen et al. [16], we can find a Multiple Access Control (MAC) protocol called Cognitive Radio Carrier Sense Multiple Access with Collision Avoidance (CR-CSMA/CA), which resolves the hidden primary terminal problem. In this protocol, an SU transmitter first conducts carrier sensing to avoid transmission overlap, and then executes a mutual spectrum sensing operation to protect the hidden PU receiver. In mutual spectrum sensing, an SU transmitter synchronizes with the corresponding SU receiver via a control packet in order to continue their respective sensing operations at the same time. Therein, both transmitter and receiver can simultaneously determine the channel’s status in their respective sensing zones. To this end, the transmitter broadcasts a new packet called Prepare-To-Sense (PTS) to let the SU receiver continue mutual spectrum sensing. Therein, the SU transmitter and SU receiver confirm the silence of PUs at the same time. Later, both SUs exchange the Request-To-Send (RTS) and Clear-To-Send (CTS) packets to ensure DATA packet transmission as part of the classic CSMA/CA [17]. However, this protocol is not very attractive to the IoT environment due to the additional overhead from the PTS packets.

For CR-based IoT networks, IoT technology also brings intrinsic key issues, which include low power consumption, high throughput, more mobility, and extensive scalability [18]. Unfortunately, most of the existing technologies and protocols do not fully support IoT applications. For example, Low Power Wireless Personal Area Network (LP-WPAN) technologies (e.g., ZigBee, Bluetooth LE, and 6LoWPAN) for short-range communications support a few hundred kilobits per second. On the other hand, Low Power Wide Area Network (LP-WAN) technologies (e.g., SigFox and LoRA) for long-range communications only support a few kilobits per second. The short range of WPAN and low throughput of LPWAN technologies limit their application in the IoT.

Moreover, although the prevalent IEEE 802.11 standard achieves better performance for Wireless Local Area Networks (WLANs), it lacks support for super-dense IoT networks. Actually, stations in WLANs under the super-dense environment create frequent collisions that not only cause high power consumption but also degrade throughput. Rango et al. [19] estimated that wireless stations may consume 50% of their total energy due to collisions at high load by super-dense WLANs. Akella et al. [20] obtained measurement results in several cities to prove that more than ten closely deployed Service Access Points (SAPs) for IEEE 802.11 networks cause severe collisions with each other. This situation will get worse if more SAPs are deployed to act as contenders.

Given the issues mentioned above, the IEEE 802.11 Task Group initiated the IEEE 802.11ah project to enact a standard that can operate on sub-1 GHz license-exempt bands [21]. The major objectives of this project are allowing a large number of stations within wide range of a network, but with a high data rate and low power consumption. MAC protocol in IEEE 802.11ah is based on placing a number of participating stations into multiple groups to reduce the number of collisions. The stations in each group are alternately allowed access to the channel in a Restricted Access Window (RAW) that lasts for a limited period of time. The designated stations in the RAW use the traditional Enhanced Distributed Channel Access (EDCA) to access the channel [22]. Meanwhile, stations outside the RAW do not participate, but they do go into sleep mode and save energy. The participating stations determine their RAW through coordination with the SAP for uplink or downlink transmissions.

IEEE802.11ah assumes that every station can directly connect with the SAP. However, this assumption is not always true, since SAP services are not always available to the serving stations. Moreover, SAPs require signaling overhead to control the groups of stations. For the SAP, more regroupings are required due to frequent variations in the number of participating stations over time, and they thus incur more overhead [22]. This phenomenon is likely to be common in IoT networks with mobile devices like vehicles and mobiles phones, and such networks are usually hampered by the side effects of mobility [23]. Similarly, if the SAP somehow fails, it will stop the entire network from serving clients, and will disrupt all the transmissions. The stations should, therefore, be capable enough to develop an ad hoc network in an IEEE 802.11ah system.

The wide application of ad hoc networks provides motivation for IoT realization from ease of deployment, self-organization, and cost-effectiveness. Therefore, research room exists for CR-based decentralized IoT networks. To this end, a grouping strategy like that in IEEE 802.11ah can sufficiently resolve the contention problem in a super-dense network. However, the grouping of stations in decentralized networks can incur the rendezvous issue. That is, suppose that the stations are grouped and that each group is assigned to a different RAW. Then, a transmitter and its receiver might be positioned in different groups and cannot communicate with each other. On the other hand, this problem does not occur with the SAP under IEEE 802.11ah, since the SAP is the only receiver for all transmitters. Furthermore, the number of stations in the network is not known in the decentralized setup. Under such a scenario, defining the groups and their sizes becomes crucial in order to fix the number of RAWs required at any moment. However, with centralized networks like IEEE 802.11ah, such problems do not exist, since the number of connected stations is always known by the SAP.

In CR networks, channel access can either be controlled by spectrum sensing [24] or by a geolocation database method [25]. In spectrum sensing, the SU can autonomously detect the activity of PUs with energy detection or cyclostationary feature detection. Conversely, with a geolocation database, a centralized architecture is required to communicate white space information to SUs. It is debatable as to which of the two methods is suitable to ensure channel access and measure interference levels. However, when we talk about a decentralized setup, the former can be preferred over the latter due to low cost and ease of implementation [26]. For example, in IEEE 802.11af-based licensed TV bands, plenty of white space exists but with a limited number of broadcast stations [27]. Therefore, those broadcast stations cannot fully deliver local information on white space to the SUs at any one given time and location. Otherwise, more broadcast stations must be installed to protect the PUs. If stations of unlicensed networks, e.g., IEEE 802.11ah, can enable spectrum sensing in primary networks, e.g., IEEE 802.11af, then SUs can simply identify the local white space without coordination with broadcast stations.

In this paper, we propose a new decentralized MAC protocol for CR-based IEEE 802.11ah networks, in which users of the IEEE 802.11ah standard can dynamically access the TV bands of IEEE 802.11af networks as SUs. Our protocol is called carrier sense Restricted Access with Collision and Interference Resolution (RACIR), since it features carrier sensing with spectrum sensing for scalability and interference resolution. Unlike wireline networks, channel sensing is usually not feasible during data transmission in wireless networks due to the deafness problem. Therein, the receiver of the transmitting node is overwhelmed by its own transmission power. To this end, the design of the proposed protocol was inspired by Wireless-CSMA/CD [28] and CSMA/CR [29], since they characterize collision detection and collision resolution in wireless networks, respectively. We use a hybrid approach to Wireless-CSMA/CD and CSMA/CR with a novel application of interference resolution in CR-based decentralized IoT networks.

The key contributions of this paper are summarized as follows:

- We propose a novel MAC protocol for CR-based IEEE 802.11ah networks that resolves both the scalability issue and the hidden primary terminal problem.
- We develop a new decentralized algorithm that estimates the number of participating stations in the network to judicially organize them into different groups.
- We analyze the normalized throughput of our proposed protocol with the Markov chain model and compare the results with that of CR-CSMA/CA. We also compute analytic expressions to evaluate the performance of the proposed MAC in terms of average packet delay and average energy consumption per delivered bit.

The remainder of the paper is organized as follows. Section 2 provides an overview of the IEEE 802.11ah standard. Section 3 similarly takes an overview of IEEE 802.11af standard. Section 4 summarizes related work. Section 5 presents the system model. Section 6 describes the proposed MAC protocol, and Section 7 analyzes it through a mathematical model. Section 8 validates our mathematical model and discusses the results. Finally, in the last section, we summarize the paper and draw conclusions.

## 2. IEEE 802.11ah Standard Overview

The IEEE 802.11ah standard [21], to be named Wi-Fi HaLow, was published in March 2017. Under this standard, a single SAP is authorized to serve a maximum of 8191 stations within a radius of 1 km. The minimum data rate the SAP can support in IEEE 802.11ah systems is 100 kbps. Good channel propagation characteristics of unlicensed sub-1 GHz bands like long coverage, high data rates, and large scalable operations make IEEE 802.11ah unique compared to traditional WLANs. We briefly describe the IEEE 802.11ah systems in the following subsections.

### 2.1. Physical Layer

The Physical (PHY) layer of the IEEE 802.11ah system is designed on Orthogonal Frequency Division Multiplexing (OFDM) modulation method and is the down-clocked version of the IEEE 802.11ac system. The supported antenna technologies are Multiple Input Multiple Output (MIMO), downlink multi-user MIMO, and single-user beamforming. The IEEE 802.11ah system defines multiple channels with different bandwidths: 1 MHz, 2 MHz, 4 MHz, 8 MHz, and 16 MHz, since the channelization specifications of sub-1 GHz bands are different in different countries. For example, in South Korea 917.5–923.5 MHz with bandwidths of 1 MHz, 2 MHz, and 4 MHz are available, and in the United States, 902–928 MHz with bandwidths of 1 MHz, 2 MHz, 4 MHz, 8 MHz, and 16 MHz are available [30]. It is, therefore, IEEE 802.11ah that considers channelization to support the respectively available spectrum in various countries, including the United States, Europe, South Korea, China, Japan, and Singapore [31]. As an extension to this, the respective Modulation and Coding Schemes (MCSs) are also defined to support the different data rates. For instance, MCS0 provides data rates between 650 kbps and 7.8 Mbps with a 2 MHz channel.

### 2.2. MAC Layer

The MAC layer under IEEE 802.11ah is designed to support a large number of stations while ensuring minimal power consumption. In this regard, the tailored features of IEEE 802.11ah MAC are briefly discussed in the following.

#### 2.2.1. Organization of Associated Stations

**Figure 2:**
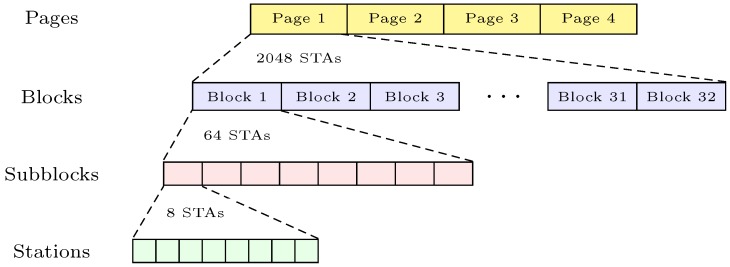
IEEE 802.11ah AID hierarchy.

The SAP of IEEE 802.11ah organizes the associated stations into a new hierarchical structure in order to support a large-scale network [32]. In a network, during the association stage, the SAP assigns a 13-bit identifier to each station, which is called the Association IDentifier (AID). The AID structure consists of four hierarchical levels: pages, blocks, subblocks, and stations, as illustrated in Figure 2. That is, a total of eight stations are indexed in a given subblock, and a total of eight subblocks are indexed in a specific block, which can contain up to 64 stations. Similarly, 32 blocks belong to a certain page, which contains up to 2048 stations. There exist four pages in each AID structure, which means that an SAP can support up to 8191 (=$2^{13}-1$) stations in a network. Using the hierarchical AID structure, IEEE 802.11ah enables four times greater support to the stations than that of the legacy IEEE 802.11 standard. Furthermore, the hierarchical AID structure also allows the SAP to group stations more easily. For example, an SAP can easily indicate multiple stations based on their block ID or subblock ID instead of their AIDs and it can also simply categorize them based on their properties such as quality of service, energy level, or type of applications.

#### 2.2.2. Channel Access and Power Saving

The stations in the IEEE 802.11ah system alternately enter awake and sleep states to save energy. In the awake state, stations keep their radio components ON, so as to continuously sense the incoming signals sent by the SAP. On the other hand, once the stations go into sleep mode, they turn their radios OFF, and cannot listen to the incoming signals during that period. Meanwhile, the SAP buffers the packets destined to a station in the sleep state until that station wakes up and requests delivery of those packets. The SAP periodically broadcasts a beacon frame, which includes a Traffic Indication Map (TIM) Information Element (IE) for the stations in a sleep state. The partial virtual bitmap field of the TIM IE in the beacon frame informs each station about the existence of the buffered packets. Such stations are organized into TIM groups and are called TIM stations, since they are obligated to listen to their respective TIM beacon. Therefore, TIM stations wake up periodically to receive the beacon frame and keep themselves up to date. If a station finds there is buffered traffic for it, then it keeps waking up and sends a Power Saving (PS)-pool frame in order to ask the SAP for delivery of the buffered traffic. Otherwise, it simply returns to the sleep state until the anticipated time of the next beacon frame.

**Figure 3:**
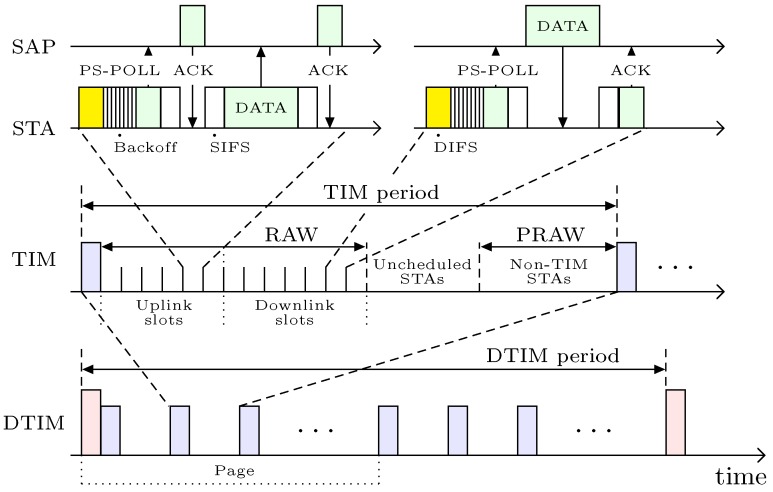
IEEE 802.11ah access mechanism.

IEEE 802.11ah splits the time scale into non-overlapping pages, Delivery TIM (DTIM), TIM and slot, as illustrated in Figure 3. DTIM and TIM periods begin once their respective beacon frames are sent by the SAP. The time duration between two consecutive DTIM beacon frames is the length of a DTIM period. Similarly, the length of a TIM period is the time interval between two consecutive TIM beacons. The DTIM-beacon is designed for multicast and broadcast traffic. This is so the group of stations (or TIM group) that belongs to the same TIM period is informed about the buffered packets waiting at the SAP. Conversely, the TIM-beacon is designed for unicast traffic such that a TIM group is informed as to which TIM station has buffered packets at the SAP. In a DTIM period, multiple TIM beacons exist to separately organize the multiple TIM groups. In addition, multiple TIM periods can be further organized into pages, which can eventually increase the number of TIM groups in each DTIM period. With multicast (or broadcast) traffic, the SAP starts transmission of the buffered packets to the allocated TIM groups after transmission of the DTIM beacon. Hence, the power saving stations in those TIM groups wake up for the expected transmission of the DTIM beacon to receive the corresponding multicast (or broadcast) packets.

In a classic IEEE 802.11 system, one drawback is that, essentially, all stations wake up to listen to the beacon frame. Accordingly, the partial virtual bitmap field of a TIM IE in the beacon is increased to an unaffordable length, especially when the size of the network is large. Ultimately, stations inevitably wake up simultaneously and wait for a long time to receive their buffered traffic due to the increased number of collisions. Moreover, if the amount of buffered traffic is also large, then TIM stations have to stay awake for a long time and consume more energy. However, the segmentation mechanism in IEEE 802.11ah splits the partial virtual bitmap field of the TIM IE within multiple beacon frames for non-overlapping periods. This prevents the power-saving stations of different TIM groups from simultaneously contending for the channel. In this mechanism, TIM stations do not have to wake up to listen to every TIM beacon; they only wake up to receive the beacon for the group they belong to. Meanwhile, stations just contend for a channel within the single TIM group to possibly ensure fewer collisions.

Under IEEE 802.11ah, another power saving and access mechanism exists for stations that require channel access occasionally. These stations are known as non-TIM stations, since they do not need to wake up to listen for a TIM-beacon. Normally, non-TIM stations negotiate a Target Wake Time (TWT) with the SAP during the association phase, and so manage to keep a long sleep mode. The power-saving non-TIM stations wake up at the TWT to listen to the Null Data Packet (NDP) paging frame. The NDP frame contains the target wake time, and a partial virtual bitmap field of the TWT IE, which further informs non-TIM stations about the status of the buffered traffic. The information in the TWT IE also temporally disperses the non-TIM stations to avoid collisions. Thus, only the allocated non-TIM station contends to access the channel within a Periodic RAW (PRAW) through the EDCA mechanism [33]. In a PRAW, multiple slots also exist for uplink or downlink transmissions. The winner of each slot sends a PS-pool message to the SAP for delivery of the buffered traffic when it identifies the buffered status. Otherwise, it may utilize the winning slot by transmitting an uplink frame to the SAP. Furthermore, stations other than TIM and non-TIM stations are called unscheduled stations. These stations do not need to listen to a beacon or NDP frame. However, at any time, they can send a PS-poll to the SAP on an immediate-basis to request the channel. These stations transmit data irregularly, and so undertake sleep and wake states without any prior schedule.

The channel access procedure of TIM, non-TIM or unscheduled stations for the transmission of PS-pool or uplink frame is also illustrated in Figure 3. Usually, a station performs carrier sensing during the Data Interframe Spacing (DIFS) interval to ensure the other stations are silent. When the channel is clear, the transmitter conducts a backoff process in order to avoid collisions with other stations. The winning station first sends a PS-pool frame to the SAP, either for uplink or downlink transmission. In the PS-pool frame, the Uplink Data Indication (UDI) filed indicates to the SAP the existence of an uplink frame from the station. Therefore, when a station has an uplink frame, it will send a special PS-pool frame with UDI field value equal to 1. If the SAP has a free slot, it will then reply with an Acknowledgment (ACK) frame that allows the transmitter to send the data. Once the SAP receives the data, it will send an ACK to the station as a token of successful transmission. Conversely, a station does not need to include the UDI filed in the normal PS-pool frame of a downlink transmission. After receiving the normal PS-poll frame, the SAP sends buffered data to the station and thereafter, receives its ACK from the station.

#### 2.2.3. Extended Parameters

The IEEE 802.11ah system does not consider backward compatibility because it does not share any common frequency band with the classic IEEE 802.11a/b/n/ac systems. Hence, it has some more liberally introduced enhancements in various parameters in order to improve system throughput. In this connection, IEEE 802.11ah uses a more compact MAC header of 18-bytes instead of the existing 48-byte header, in which MAC addresses are replaced by the AID addresses. By this enabling process, the MAC header and the Frequency Check Sequence (FCS) field are not required by the several NDP frames like ACK, CTS, and PS-pool in the IEEE 802.11ah system. Thus, the 802.11ah system only includes a PHY header in all those NDP frames to curtail their length. Moreover, a fast frame-exchange operation is introduced in which a station can acknowledge successful transmission along with the data frame instead of transmitting an ACK to the SAP. In this operation, the gap between each transmission is reduced to a Short Interframe Spacing (SIFS) interval instead of a DIFS interval, which in part also expedites the frame exchange procedure for a station whenever it has data to send, in addition to the receive purpose. In IEEE 802.11ah, the backoff slot time and interframe space parameters are, however, increased due to the long propagation delay for its large coverage [34]. Table 1 summarizes the increased timing parameters of IEEE 802.11ah, compared to other systems.

## 3. IEEE 802.11af Standard Overview

The IEEE 802.11af standard, also known to as Super Wi-Fi (or White-Fi), was released in February 2014 [35]. Therein, PHY layer supports MIMO and multi-user MIMO operations and modulation method is OFDM as that in IEEE 802.11ac. In this standard, TV stations (or/and wireless microphones) can operate on 54 and 790 MHz VHF and UHF licensed bands. This is a first standard that liberates unlicensed users to dynamically access the white spaces on licensed channels. Under this standard, a geolocation database is maintained to regulate spectrum sharing between licensed and unlicensed users in each regulatory domain. That is, the unlicensed users query the regional database for white space information at one given time, frequency and position, to access the channel. This standard has an attraction to the wireless transmission services operating at TV bands due to the advantage of lower path loss compared to that on ISM bands [36]. Furthermore, the non-line-of-sight coverage of TV bands makes this standard more attractive because its lower frequencies can present lower material abstraction [37]. Even so, its biggest challenge is how to limit the interference to the neighboring incumbent users during spectrum access by the unlicensed users.

## 4. Related Work

Under IEEE 802.11ah, the SAP allocates groups of stations to RAW-periods based on time division. Some researchers suggested an analytic model that evaluates system performance of the RAW method in terms of throughput, delay, and energy efficiency [38,39]. Yoon et al. [34] evaluated the impact of the hidden terminal problem in the IEEE 802.11ah system and proposed a regrouping algorithm to alleviate its effects on system performance. Hzmi et al. [40] presented some holding schemes for RAW handover between the groups in order to improve throughput and energy efficiency. Under the holding schemes, stations essentially hold themselves to continue transmission before crossing the boundary of their allocated RAW-period to avoid collisions. Kim et al. [41] determined that, based on time division, grouping of stations is likely to degrade system performance. This is because an inevitable holding period is required during the handovers between the groups. For that, they suggested that the grouping strategy should be based on transmission attempts in order to avoid wasting the channel. That is, a group of stations within a RAW-period is allowed to utilize slots based on the number of successful attempts, instead of the fixed length of the RAW-period, so that stations in the last slot do not need to hold their transmissions before the expiry of allocated RAW-periods.

We found more research that specifically focused the RAW mechanism on various performance measures [42,43,44]. Zhao et al. [42] optimized the performance of the RAW-mechanism in terms of power consumption, and showed that energy efficiency in the sensor nodes improves with an increasing number of RAW-groups. Tian et al. [43] evaluated the performance of RAW-grouping parameters under a variety of network configurations and highlighted the need for adaptation of grouping parameters to improve network efficiency. Tian and colleagues [44] suggested an optimization algorithm to judicially define the grouping-parameters based on real-time traffic, and improved network efficiency.

We can classify grouping strategies into centralized and decentralized schemes. For example, Chang et al. [45] proposed a load-balanced grouping algorithm to form efficient groups of sensors that are connected to the SAP server. Other researchers proposed analytic models to track throughput performance of a centralized grouping strategy under a super-dense network [23,46]. In addition, we found some other so-called decentralized grouping schemes [23,41], but they did not consider a decentralized topology in the network. However, all these schemes assumed that there always exists a centralized receiver (or SAP), which coordinates all the stations in terms of channel access, power control, sleep schedule, and other RAW parameters. We found relay-based schemes for IEEE 802.11ah [47], in which the relay stations, connected to the root-SAP, can act as relay-SAPs. However, those authors did not consider the implementation issues of the voluntary root-SAP services like the selection process of the relay-stations and their energy consumption. In that case, the relay stations can specifically drain energy very quickly due to the overhead of coordination with the root-SAP and a large number of stations.

Another approach exists to reduce collisions among stations, which can be called a contention alleviation scheme [48,49]. In this scheme, stations purposefully enlarge the contention window (*W*) in order to reduce the collision probability. Typically, stations doubled the size of the window (until a maximum value) whenever a collision is observed in an IEEE 802.11 system. If stations set the size of the window too small, they suffer from collisions more frequently. Conversely, if the size of the window is too large, stations likely reduce collisions but system throughput is likely reduced due to larger access delay. To this end, Bianchi [49] determined that the optimal value of the window is a function of the total active stations in the network such that $W_{opt}=\sqrt[{n}]{2T}$, where *n* is the number of stations attempting to access the channel, and *T* is the transmission time of a data frame. However, this scheme may not be practical in super-dense networks due to the larger access delay for the stations. For instance, 8000 stations will at least have an optimum value for a window larger than 16,000 backoff slots, which will extremely increase access delay. Eventually, the efficiency of the system will also be compromised.

In typical ad hoc networks, clustering [50,51] is another idea in order to implement grouping. In clustering schemes, stations are organized into multiple clusters based on their geographic locations, and a cluster head often coordinates communications between stations through an exchange of location information messages. A detailed study on clustering schemes for ad hoc networks was done by Yu and Chong [52]. The major difference in the grouping (as with that in IEEE 802.11ah and clustering) is that grouping utilizes the time dimension, while clustering exploits the space dimension of the spectrum [23]. Therefore, the benefits of clustering are limited in a super-dense network where stations are most likely close to each other. In such a scenario, more location information messages will be required to maintain clusters under a deep hierarchy, which cannot only drive stations to drain more energy but can also consume bandwidth at a large scale.

## 5. System Model

**Figure 4:**
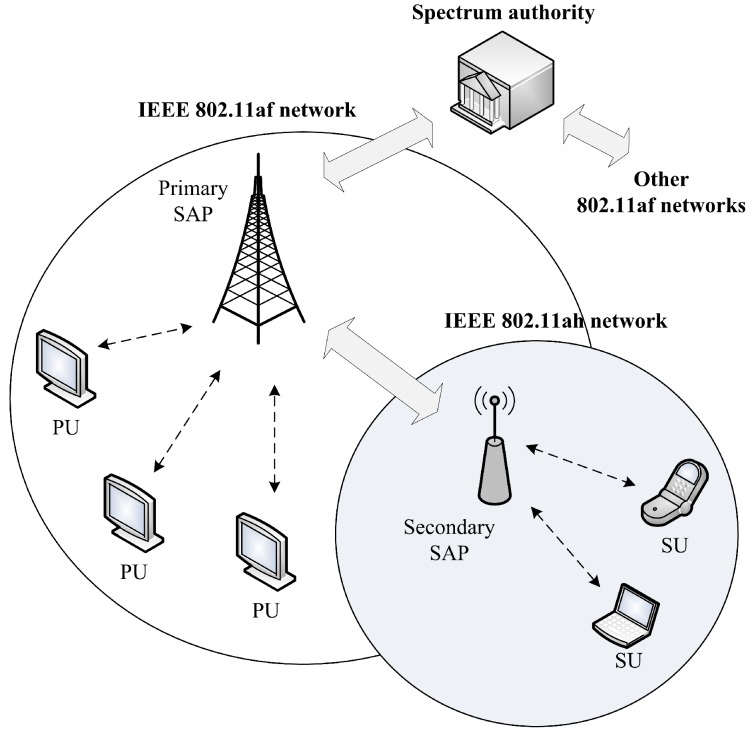
System model.

We consider *N* SUs in a secondary network, which are co-located with multiple PUs in a primary network. SUs and PUs, respectively, belong to IEEE 802.11ah and IEEE 802.11af networks, as shown in Figure 4. There exists no collaboration between PUs and SUs because both primary and secondary networks operate non-cooperatively. However, SUs can communicate with each other in a self-organized decentralized network without the SAP server. SUs can only rely on the SAP for Internet services when they act as IoT devices. SUs can occupy the licensed channel whenever all the neighboring PUs are inactive. Meanwhile, if a neighboring PU is active, SUs are obligated to vacate the channel immediately. We assumed an error-free single channel model, in which packet loss takes place only due in part to the collisions between SUs and/or interference with PUs. Each SU conducts spectrum sensing to detect the activity of PUs in its neighborhood. SU *j* can detect the neighboring PUs as active with a probability of $\pi_{1,j}$, and as inactive with a probability of $\pi_{0,j}$.

In a real environment, perfect sensing is a big challenge. Thus, there always exists a certain probability of misdetection and false alarm. For SU *j*, we denote the misdetection probability as $\alpha_{j}$ and the false alarm probability as $\beta_{j}$. Misdetection indicates that an SU mistakenly recognizes active PUs as idle, which leads to significant interference due to subsequent transmissions by SUs. When there are *N* SUs, the false alarm probability increases as seen on the left side of Equation (1), assuming that misdetections between users are independent:

(1) $$1-{\left(1-\alpha_{j}\right)}^{N}\leq {\hat{\alpha }}_{j}.$$

We require that the misdetection probability be bounded by a predefined value $\hat{\alpha }$. On the other hand, a false alarm means that an SU mistakenly detects an idle PU as active. Then, SUs can miss a transmission opportunity. The false alarm probability depends on sensing time *T* [53] as follows:

(2) $$\beta_{j}=Q\left(\sqrt{2\gamma +1}Q^{-1}\left(1-\alpha_{j}\right)+\sqrt{T_{fs}}\gamma \right),$$

where γ is the Signal-to-Interference-and-Noise-Ratio (SINR) of the PUs’ signals measured at SU *j*, $f_{s}$ is the sampling rate of the channel, and Q(·) represents the complementary distribution function of a standard Gaussian variable.

## 6. Proposed MAC Protocol

The proposed MAC protocol, called carrier sense Restricted Access with Collision and Interference Resolution (RACIR), is based on the random access model. In RACIR, system time is divided into non-overlapping and equal time slots. The SUs in the designated group can only access the shared channel at the beginning of each time slot, which is denoted as σ. The SUs are assigned to the designated group with our group split algorithm, which is explained in Section 6.4.

### 6.1. RTS/CTS Access Mechanism of RACIR

We here discuss how SUs in a designated group access a channel within the allocated RAW-period using control frames. According to RACIR, the intended SU first senses the channel for an Extended Interframe Space (EIFS) interval. Therein, if the channel is sensed as idle, it then exchanges RTS/CTS with the corresponding SU receiver, as part of CSMA/CA. The SU transmitter can start data transmission after successful exchange of RTS and CTS packets. Right after the beginning of data transmission, both SU transmitter and SU receiver start spectrum sensing for a short interval, called the Interference Detection (ID) slot. Both of the SUs randomly choose an ID slot from the ID period in order to detect the activity of neighboring PUs in their respective spectrum-sensing zones.

If a PU is found to be active by either the SU transmitter or the SU receiver, it will first broadcast a JAM signal to stop the ongoing transmission, and it then goes into a blocking state. After receiving the JAM signal, the other SU also blocks itself for a predefined period to protect the ongoing transmission of the PU. In the blocking state, the length of the blocking period is equal to twice the data transmission period. When blocking, the detection of a JAM signal for SUs nevertheless depends upon the PHY layer and the propagation delay required in switching from transmission (Tx) to receiving (Rx) or from Rx to Tx. Whenever the PU is not active and a JAM signal is not received, the SU transmitter will continue transmitting data packets, and will then receive an ACK from the corresponding receiver to complete the transmission. The length of the ID slot is of necessity shorter than the EIFS interval so that other SUs do not interfere. Furthermore, it should be greater than the JAM signal’s detection time plus the switch time for Tx to Rx or Rx to Tx, to let the SU invoke the blocking state. We mention that PUs can exercise transmission priority rights compared to SUs due to the large interval of the EIFS in carrier sensing and application of a JAM signal after the mutual spectrum sensing operation.

### 6.2. Basic Access Mechanism under RACIR

For the one-hop distance of a fully connected network, our RACIR protocol is also applicable to the basic access mechanism as standardized by IEEE 802.11. In the basic access mechanism for RACIR, SUs in competition must first sense the channel as idle for the EIFS interval. Then, they individually choose a backoff counter to avoid collision. The SU transmitter where the backoff counter expires first reserves the right to transmit data packets directly. Right after data transmission begins, it conducts mutual spectrum sensing to enable the interference resolution process, similar to the RTS/CTS access mechanism in RACIR. Conversely, the other SUs decrease their backoff counters one by one whenever the channel is found idle for the EIFS interval again.

**Remark**: In our proposed MAC, if a PU becomes active during the exchange of control packets, the PU interferes until the completion of RTS/CTS transmission. This interference is acceptable in CR networks in the following senses:

- IEEE 802.22, a representative standard in CR systems, requires the SUs to vacate the channel within 100 ms when PUs become active [54]. It is enough for completion of an RTS/CTS operation.
- The activity rate of PUs is very low in the cognitive radio environment [2,3,4]. Hence, it is not likely for PUs to be active more frequently.

However, in the ID slot, we have considered a spectrum sensing technique [29], such as energy detection, cyclostationary feature detection, etc., with a JAM signal to resolve interference with the PU during data transmission.

### 6.3. PU Detection and Protection via JAM Signal

The length of the ID slot, the ID period, and detection of a JAM signal must still be designed. In this connection, let us take an analogy of the Clear Channel Assessment (CCA) method, as defined by the IEEE 802.11 standard. The CCA method in the standard evaluates the channel as busy either by carrier sensing or spectrum sensing [17]. Therein, aSlotTime is the firm time required to detect a carrier signal in the PHY layer in various modes, e.g., Direct Sequence Spread Spectrum (DSSS), Frequency Hopping Spread Spectrum (FHSS), etc., in order to access the channel, which is given as:

(3) $$\begin{array}{c} aSlotTime=aCCATime+aRxTxTurnaroundTime\\ +aAirPropagationTime+aMACProcessingDelay, \end{array}$$

where aCCATime is the required clear channel assessment time by the PHY layer, aRxTxTurnaroundTime is the maximum time required to switch from receive to transmit states, aAirPropagationTime is the maximum time required for a signal to travel to its destination, and aMACProcessingDelay is the time required by the MAC to issue a request to the PHY layer.

For our RACIR protocol, we can develop a distinguishable pattern for the JAM signal similar to that of the preamble in the IEEE 802.11 standard. Therefore, the detection of JAM signal within aCCATime can be possible. Our RACIR protocol, however, requires a Tx-Rx-Tx transition to complete a spectrum sensing operation during data packet transmission. To ensure this, a Tx/Rx turnaround time is desirable for inclusion in aSlotTime, as defined in CSMA/CR [29]. The length of the ID slot, therefore, includes aSlotTime+aTxRxTurnaroundTime. In addition, an ID slot time should be shorter than the EIFS interval, which is defined by the IEEE 802.11 standard as follows:

(4) $$\begin{array}{cc} EIFS= & aSIFSTime+aDIFSTime+(8\times ACKSize)\\ & +aPreambleLength+aPLCPHeaderLngth, \end{array}$$

where (8×ACKSize) is the length of an ACK frame in bytes and aPLCPHeaderLngth is the PHY header length expressed in microseconds. Hence, an ID slot (aIDSlotTime) must satisfy

(5) $$\begin{array}{cc} aSlotTime+ & aTxRxTurnaroundTime\\ & \;\leq aIDSlotTime<EIFS. \end{array}$$

Lastly, the slot period length can readily be set as the multiples of ID slots such that aIDSlotPeriod=8×aIDSlotTime.

### 6.4. Group Split Algorithm

It is not easy to estimate the number of stations during a normal channel access operation since it is very complicated. Whenever we want to estimate the number of stations, we let each station behave in a predefined simple way, which enables probabilistic analysis. To this end, we develop a purpose-built group split algorithm that estimates the number of active stations in the network. The key idea is to split the number of stations into multiple groups based on the current size of the network. If the number of SUs in a designated group is too large for the slots in an allocated RAW-period, the efficiency of the network may go down due to a large number of collisions. Conversely, if the number of SUs in a designated group is fewer than the slots in an allocated RAW-period, the efficiency of the network could also be compromised due to empty slots. We, therefore, design the following group split algorithm.

Any arbitrary station broadcasts a packet announcing the start of the estimation phase. The packet carries a probability parameter *a*, and a target group size $n_{0}$. After receiving the packet, each station can access the following *estimation slot* with a probability of $1-a^{1/n_{0}}$ independently for each estimation slot. Thus, no station accesses an estimation slot with probability $a^{N/n_{0}}$, where *N* denotes the number of stations that we want to estimate. Suppose that we set the number of estimation slots at *K*, and, among them, no station has accessed *k* estimation slots. Letting $\Omega =a^{N}/n_{0}$, we know that such an event occurs with a probability of

(6) KkΩk(1−Ω)K−k.

This probability is maximized at $\hat{\Omega }=k/K$ such that we can estimate $a^{\hat{N}/n_{0}}=k/K$, i.e.,

(7) $$\hat{N}=n_{0}\frac{\ln\frac{k}{K}}{\ln a}.$$

This is a Maximum Likelihood (ML) estimation, where *a* is our design parameter. If the estimated number of stations is larger than the target group size, i.e., $\hat{N}\geq n_{0}$, we split the stations into two separate groups. To this end, stations are individually liable to estimate the number of active stations at any one moment. Therefore, if the number of estimated stations is larger than the target group size, each station then chooses a random number between 0 and 1 to select a new group. Otherwise, stations do not split into groups.

## 7. Performance Analysis

We now analyze the performance of our proposed protocol in terms of normalized throughput, average packet delay and average energy consumption. For quick reference, we summarize the symbols in Table 2.

### 7.1. Packet Transmission Procedure

**Figure 5:**
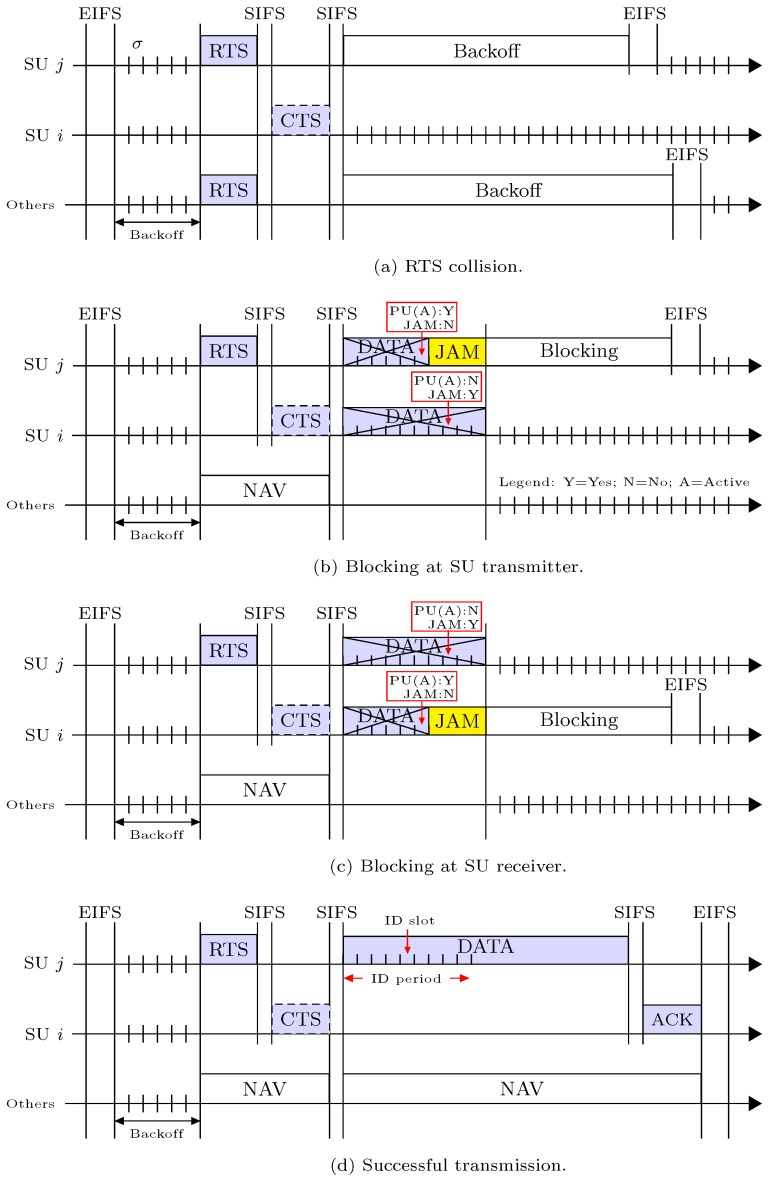
RACIR access mechanism in all possible events.

We investigate the data packet transmission procedure of the proposed MAC protocol. In the data transmission attempt of SU *j*, there exists one of the following four events: (1) RTS collision; (2) Blocking at the SU transmitter; (3) Blocking at the SU receiver; and (4) Successful transmission, as illustrated in Figure 5. We denote the four possible events by $\varepsilon_{i},i=1,\cdots ,4$. We now calculate the probability (*P*) and length of time (*T*) for each possible event. To this end, we know that SU *j* can access the medium whenever the channel is free (or clear) from the activity of PUs. We can obtain the *clear-channel* probability (κ) of SU *j* as

(8) $$\kappa_{j}=\alpha_{j}\pi_{1,j}+\left(1-\beta_{j}\right)\pi_{0,i},$$

where $\beta_{j}$ is the false alarm probability, $\alpha_{j}$ is the misdetection probability of SU *j*, $\pi_{1,j}$ is the probability of activity, and $\pi_{0,j}$ is the probability of inactivity by PUs, respectively. Hence, SU *j* decides on activity or inactivity of the PUs with probability $1-\kappa_{j}$ and $\kappa_{j}$, respectively.

Recall that our proposed MAC enables the RTS/CTS mechanism in accordance with the traditional CSMA/CA protocol. Therein, SUs with packets to send compete with each other by randomly choosing a backoff counter value first. Then, they conduct carrier sensing to determine whether the channel is clear or not. If the channel is idle for the EIFS interval, SUs then decrease the backoff counter by one whenever a backoff slot is idle. The SU with the backoff counter that expires first will ultimately win the channel. Suppose that SU *j* wins the channel and broadcasts RTS. SU *j* may, however, fail to hear CTS from the corresponding receiver because its RTS collided with another RTS, which is event $\varepsilon_{1}$. Let, τ be the probability of transmission by an SU with a non-empty queue and backoff counter 0. Hence, the RTS collision probability is given by

(9) $$P_{\varepsilon_{1}}=\kappa_{j}\left(1-\prod_{n=2}^{N}\left(1-\tau_{n}\kappa_{n}\right)\right),$$

where $\tau_{n}$ and $\kappa_{n}$ accounts for the clear-channel probability and transmission probability of SU *n*, respectively. We mention that $1-\tau_{n}$ refers to the probability when *n* SUs do not transmit due to an empty queue. Hence, the second term of the equation indicates that at least one RTS of *n* SUs, i.e., n=2,…,N, collides with SU *j*’s RTS. From Figure 5a, the length of time spent by SU *j* due to event $\varepsilon_{1}$ can be written as

(10) $$T_{\varepsilon_{1}}=RTS+CTS+SIFS+EIFS,$$

where RTS and CTS denote the transmission times for RTS and for CTS, respectively.

Suppose that SU *j* has received the CTS and starts data transmission. Right after data transmission begins, it conducts spectrum sensing in a randomly chosen ID slot and detects the PUs as active. We called this event $\varepsilon_{2}$ that encounters with probability

(11) $$P_{\varepsilon_{2}}=1-\kappa_{j}.$$

Later, event $\varepsilon_{2}$ happens, and SU *j* immediately stops data transmission, sends a JAM signal to the corresponding receiver SU *i*, and switches to the blocking state. Once the JAM signal is received, SU *i* stops receiving data and then enters the blocking state. From Figure 5b, the length of time elapsed by event $\varepsilon_{2}$ is given as

(12) $$T_{\varepsilon_{2}}=RTS+CTS+SS+JAM+2SIFS+EIFS,$$

where SS and JAM, respectively, represent the spectrum sensing time and the transmission time of the JAM signal.

Suppose that SU *j* has sent RTS and in response, SU *i* replies with CTS. In the cascade, SU *j* starts transmitting data after the SIFS interval. As long as SU *i* receives the first bit of a data packet, it randomly selects an ID slot for spectrum sensing. Therein, if SU *i* detects the PUs as active, it stops receiving data, sends a JAM signal, and then switches to the blocking state. We denote this event as $\varepsilon_{3}$, in which SU *i* performs all these actions in order to protect the hidden PU receiver. Hence, we can compute the probability of event $\varepsilon_{3}$ as

(13) $$P_{\varepsilon_{3}}=\kappa_{j}\left(1-\kappa_{i}\right)\left(\prod_{n=3}^{N}\left(1-\tau_{n}\kappa_{n}\right)\right),$$

where $\kappa_{i}$ is the clear-channel probability for SU *i*. The last term of this equation indicates that none of the *n* SUs, i.e., n=3,…,N, collides with SU *j*’s transmission. From Figure 5c, the length of event $\varepsilon_{3}$’s elapsed time is

(14) $$T_{\varepsilon_{3}}=RTS+CTS+SS+JAM+2SIFS+EIFS.$$

We now consider the last event $\varepsilon_{4}$, which is successful transmission. Suppose that SU *j* and SU *i* have exchanged RTS and CTS correctly. SU *j* then starts transmitting data and performs spectrum sensing in an ID slot chosen at random. Meanwhile, SU *i* also conducts spectrum sensing and continues to receive data. In this case, both of the SUs luckily do not observe any PU activity. Therefore, SU *j* completes data transmission and positively receives ACK if there is an error-free channel. However, other SUs follow the backoff process according to the Network Allocation Vector (NAV), whose values are updated by the RTS, CTS, and ACK packets shared in the network. Hence, we can compute the probability of event $\varepsilon_{4}$ as

(15) $$P_{\varepsilon_{4}}=\kappa_{j}\kappa_{i}\left(1-\tau_{i}\right)\left(\prod_{n=3}^{N}\left(1-\tau_{n}\kappa_{n}\right)\right).$$

From Figure 5d, the elapsed time of event $\varepsilon_{4}$ is given as,

(16) $$\begin{array}{cc} T_{\varepsilon_{4}}=RTS+CTS & +SS+DATA\\ & +ACK+3SIFS+EIFS, \end{array}$$

where DATA and ACK represent transmission time of both data and ACK packets, respectively.

### 7.2. Normalized Throughput

We evaluate the performance of our RACIR protocol under the following assumptions:

- The secondary network is a fully connected single-hop environment, in which SUs are directly connected to each other with the topology of a complete graph.
- The SUs initially belong to the same group, which are later segregated into equal-sized multiple groups by the group split algorithm.
- The SUs in each group rendezvous with each other in random fashion and access the channel periodically.
- The SUs transmit control and data packets on both a single and a shared channel.
- The secondary network is saturated, in which SUs always have packets to send.

**Figure 6:**
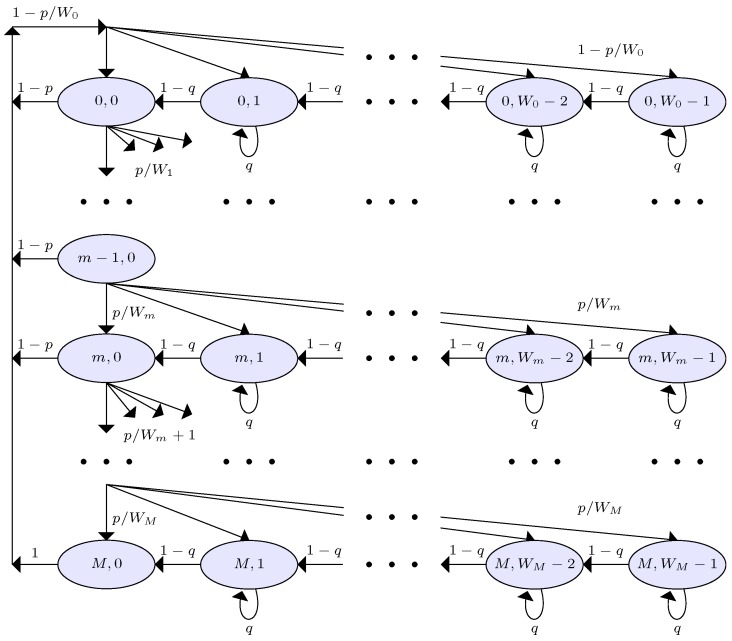
Markov chain model of the backoff procedure.

In our proposed system, we model the backoff process for SUs with the two-dimensional Markov chain model shown in Figure 6. Therein, backoff states at time *t* are the values of two stochastic processes of backoff counter b(t) and backoff stages m(t). We denote the backoff state as $S_{m,b}$, where the *m*-th row and the *b*-th column refer to the values of the backoff stage and backoff counter, respectively. The contention window size *W* at stage *m* is given as m∈[0,M], where *M* is the maximum backoff stage. However, $W_{0}$ and $W_{M}$, respectively, represent the minimum size and the maximum size of the contention window. We denote the probability of transmission failure, due to events $\varepsilon_{2}$, $\varepsilon_{3}$, and $\varepsilon_{4}$, with *p*; and the interference probability with *q*, which requires SUs to concede the blocking state. Hence, the probability of successful transmission and of non-blocking of SUs can be denoted as 1−p and 1−q, respectively.

We define interference probability *q* as a measure of PU activity, with which an SU blocks itself to protect the active PU. Please note that, if there is no false alarm and misdetection, interference probability *q* and PU activity probability $\pi_{1}$ will match exactly. For the sake of simplicity, we hence assume an ideal scenario whereby these two probabilities are equal. From Figure 6, the state transition probabilities of the Markov chain model can be written as seen in Equation (17). We observe that the Markov chain of our MAC protocol is similar to that of Chong et al. [55] due to the homogeneous and standard backoff process. From Chong et al. [55], we hence directly refer to the transmission probability of an SU *j* as Equation (18):

(17) $$\left\{\begin{array}{cc} \mathrm{Pr}\{S_{m,b}|S_{m,b}\}=q & \mathrm{for}\;m\in \left[0,M\right],\;b\in \left[1,W_{m}-1\right)\\ \mathrm{Pr}\{S_{m,b-1}|S_{m,b}\}=1-q & \mathrm{for}\;m\in \left[0,M\right],\;b\in \left[1,W_{m}-1\right)\\ \mathrm{Pr}\left\{S_{0,b}|S_{m,0}\right\}=\frac{1-p}{W_{0}-1} & \mathrm{for}\;m\in \left[0,M-1\right],\;b\in \left[1,W_{m}-1\right)\\ \mathrm{Pr}\left\{S_{m,b}|S_{m-1,0}\right\}=\frac{p}{W_{m}-1} & \mathrm{for}\;m\in \left[1,M\right],\;b\in \left[1,W_{m}-1\right)\\ \mathrm{Pr}\left\{S_{0,b}|S_{M,0}\right\}=\frac{1}{W_{M}-1} & \mathrm{for}\;b\in [1,W_{m}-1), \end{array}\right.$$

(18) $$\tau_{j}=\sum_{m=0}^{M}S_{m,0}=\frac{1-p^{M+1}}{1-p}\left(\frac{2(1-q)(1-2p)(1-p)}{W\left(1-{\left(2p\right)}^{M+1}\right)\left(1-p\right)+2\left(1-2p\right)\left(1-p^{M+1}\right)\left(1-q\right)}\right).$$

We henceforth drop the subscript *j* if there is no confusion. From Equations (9), (11) and (13), we can define the probability of transmission failure for SU *j* as

(19) $$p=P_{\varepsilon_{1}}+P_{\varepsilon_{2}}+P_{\varepsilon_{3}}.$$

Let S be the normalized throughput obtained by a secondary network. We refer to the results of Bianchi [49] for Equations (21) and (32). Let $P_{tr}$ be the probability that at least one transmission occurs when *N* SUs attempt it with probability τ

(20) $$P_{tr}=1-{\left(1-\tau \right)}^{N}.$$

Let $P_{s}$ be the probability when a given transmission by an SU is successful and the remaining N−1 transmitters defer their transmissions, provided that at least one SU transmits on a clear-channel, i.e.,

(21) $$P_{s}=\frac{N\tau {\left(1-\tau \right)}^{N-1}}{P_{tr}}=\frac{N\tau {\left(1-\tau \right)}^{N-1}}{1-{\left(1-\tau \right)}^{N}}.$$

Now, throughput S can be expressed as the following ratio:

(22) $$S=\frac{\mathrm{Average}\;\mathrm{payload}\;\mathrm{transmitted}\;\mathrm{in}\;a\;\mathrm{slot}}{\mathrm{Average}\;\mathrm{duration}\;\mathrm{of}\;a\;\mathrm{slot}}.$$

**Figure 7:**
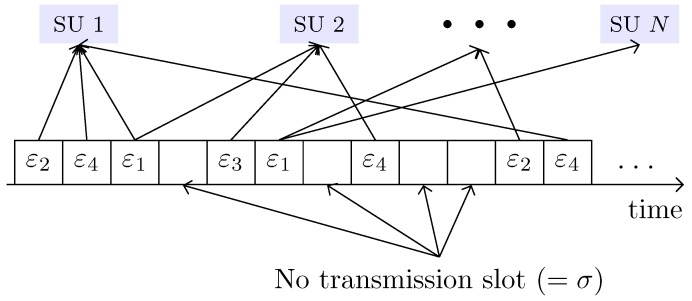
Emulation of all the possible events in a RACIR system.

Let us consider an autonomous system with an average payload E[P]. Let $P_{tr}$ be the portion of $\varepsilon_{1}$, $\varepsilon_{2}$, $\varepsilon_{3}$ and $\varepsilon_{4}$ slots, and let $1-P_{tr}$ be the portion of no transmission (or idle) slots as shown in Figure 7. $P_{tr}P_{s}$ is the portion of $\varepsilon_{2}$, $\varepsilon_{3}$, and $\varepsilon_{4}$ slots and $P_{tr}\left(1-P_{s}\right)$ is that of $\varepsilon_{1}$ slots. Therefore, the probability of SU *n*’s $\varepsilon_{i}$ slots (i=2,⋯,4) is given by

(23) $$P_{n_{i}}=\frac{1}{N}P_{tr}P_{s}\left(\frac{P_{\varepsilon_{i},n}}{P_{\varepsilon_{2},n}+P_{\varepsilon_{3},n}+P_{\varepsilon_{4},n}}\right).$$

$\sum_{n=1}^{N}P_{n_{4}}T_{\varepsilon_{4}}E\left[P\right]$ is the average amount of payload transmitted in each slot. The average duration of a slot can readily be computed considering that, with a probability of $1-P_{tr}$, the system slot time remains idle; with a probability of $P_{tr}\left(1-P_{s}\right)$, it holds as collision; probability $\sum_{n=1}^{N}P_{n_{2}}$ indicates blocking at the transmitter; $\sum_{n=1}^{N}P_{n_{3}}$ indicates blocking at the receiver; and probability $\sum_{n=1}^{N}P_{n_{4}}$ is successful transmission, which is given as

(24) $$\begin{array}{cc} E\left[R\right]= & \left(1-P_{tr}\right)\sigma +P_{tr}\left(1-P_{s}\right)T_{\varepsilon_{1}}\\ & \;+\sum_{n=1}^{N}P_{n_{2}}T_{\varepsilon_{2}}+\sum_{n=1}^{N}P_{n_{3}}T_{\varepsilon_{3}}+\sum_{n=1}^{N}P_{n_{4}}T_{\varepsilon_{4}}. \end{array}$$

Hence, Equation (22) becomes

(25) $$S=\frac{\sum_{n=1}^{N}P_{n_{4}}T_{\varepsilon_{4}}E\left[P\right]}{E[R]}.$$

### 7.3. Average Packet Delay

Let D be the average delay that a data packet incurs after leaving the source, becoming Head-Of-Line (HOL) in the MAC queue until it reaches its destination successfully. We can intuitively compute the average delay of a packet as

(26) $$D=E\left[X\right]+E\left[R\right]+E\left[T_{\varepsilon_{4}}\right],$$

where E[X] refers to the average number of slot times that an arbitrary packet observes before its successful transmission, and $E[T_{\varepsilon_{4}}]$ denotes the average length of a successful transmission. We have already calculated the average duration of a slot, i.e., E[R], in Equation (24). Hence, we need to calculate the remaining terms. From Equation (16), we can readily see that

(27) $$\begin{array}{ccc} E[T_{\varepsilon_{4}}] & =RTS+CTS & +SS+DATA\\ & & +ACK+3SIFS+EIFS\\ & =RTS+CTS & +SS+(H+E[P])/E\\ & & +ACK+3SIFS+EIFS, \end{array}$$

where *H* and *E* denote the size of the PHY and MAC headers and the channel’s bit rate for transmission, respectively.

We now calculate the average length of backoff slots as

(28) $$\begin{array}{cc} E[X] & =\sum_{m=0}^{\infty }e^{m}\left(1-e\right)\left(\sum_{k=0}^{m}\frac{W_{k}-1}{2}\right)\\ & =\left(1-e\right)\sum_{m=0}^{\infty }\left(\frac{W_{m}-1}{2}\sum_{k=0}^{\infty }e^{k}\right)\\ & =\frac{W_{0}}{2}\left(\frac{1-{\left(2e\right)}^{M+1}}{1-2e}+{\left(2e\right)}^{M+1}\frac{e}{1-e}\right)-\frac{1}{2(1-e)}, \end{array}$$

since

(29) $$W_{m}=\left\{\begin{array}{cc} 2^{m}W_{0}, & m\leq M\\ 2^{M}W_{0}, & m>M. \end{array}\right.$$

where *e* refers to the transmission error probability, such that e=p+q. We mentioned that E[X] applies to those frames for which acknowledgment was received. That is, dropped packets are not considered in order to derive the delay.

### 7.4. Average Energy Consumption

Let E be the average energy consumption per delivered bit under the proposed MAC protocol, in which stations can often consume energy during the sleep and wakeup operations. Thus, the per-bit energy consumption of a station can be written as

(30) $$E=E_{s}+E_{w}+E_{t},$$

where $E_{s}$ and $E_{w}$ refer to the energy of a station consumed during the sleep and wakeup states, respectively. Furthermore, $E_{t}$ accounts for the energy consumed in the transition state to the sleep (or wakeup) state of a station. Usually, the energy consumed during the sleep and transition states is constant. However, the energy consumption in the wakeup state is variable due to the variety of the operations. We hence only analyze the energy consumption of the wakeup state as

(31) $$E_{w}=E_{g}+E_{r},$$

where $E_{g}$ and $E_{r}$ denote the energy consumption due to the grouping and MAC operations in a RAW-period, respectively. We can find the energy consumption of a station for the grouping operation as the function of G−1, where *G* refers to the number of groups characterized by a station. On the other hand, the average per-bit energy consumption during a RAW-period is given as

(32) $$E_{r}=\frac{P_{wr}\left(S\times E\left[R\right]\right)}{RTS_{s}+CTS_{s}+E\left[P\right]+ACK_{s}},$$

where $P_{wr}$ is the transmission/reception power of a station, and *S* denotes the number of slots in a RAW-period. We recall that E[R] is the average length of a slot as derived in Equation (24), and E[P] is the average size of the payload in terms of delivered bit. Furthermore, $RTS_{s}$, $CTS_{s}$ and $ACK_{s}$ denote the sizes of RTS, CTS and ACK packets, respectively.

## 8. Results and Discussion

We have analyzed the performance of our proposed protocol with RTS/CTS and Basic access mechanisms and compared it to that of CR-CSMA/CA [16] under the system of interest. In our analytic model, we set the number of SUs *N* at 12,000 and the number of RAW-periods at 140. In each RAW-period, we set the number of RAW slots at 10 and the target group size $n_{0}$ at 100, unless otherwise stated. The optimum value of probability parameter *a* is chosen from the range 0 to 1 with the trial-and-error method. Initially, each SU estimates the network size according to the group split algorithm in order to choose a group index. Thereafter, SUs of each group, RAW-period-wise, access the channel in round-robin fashion. However, the RAW slots in each RAW-period are accessible according to the proposed protocol. We developed Monte Carlo simulations with Matlab to verify the analytic results. Furthermore, we obtained the results from averaging 1000 runs. To put it in a nutshell, we summarize the default system configurations in Table 3.

**Figure 8:**
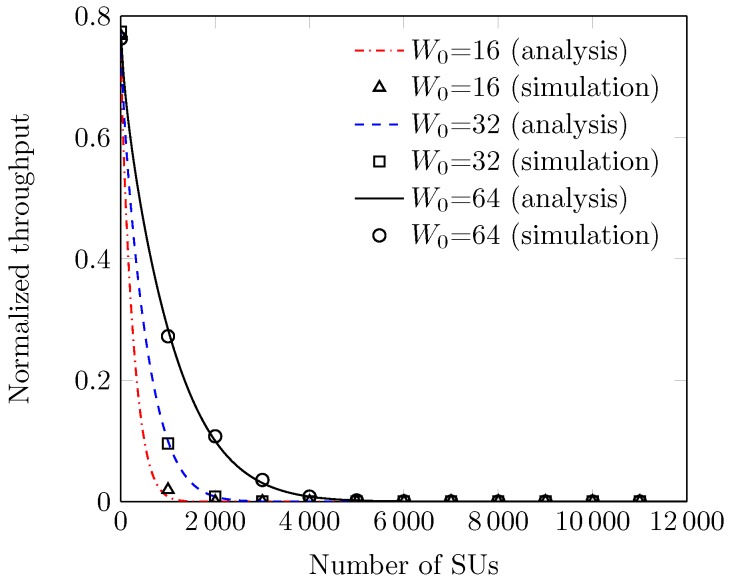
Throughput without grouping the stations.

**Figure 9:**
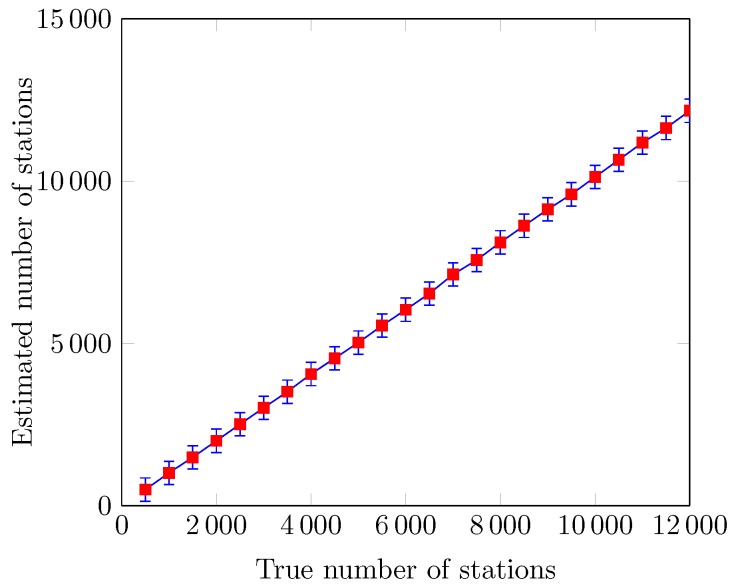
Estimated number of stations vs. true number of stations.

We here refer to the variants of the proposed protocol as RACIR-basic and RACIR-rts/cts for the Basic and RTS/CTS based access mechanisms, respectively. Figure 8 shows the normalized throughput S of the proposed RACIR-rts/cts protocol without grouping of the stations in a super-dense environment. We can see that the values of S sharply decrease with an increase in the number of SUs. This suffices to explain that the length of the maximum backoff counter from $W_{0}$ quickly becomes smaller, compared to the number of SUs, which increases the number of collisions. The channel time is being wasted and thus decreases system performance. We estimate the size of the network in order to implement the group-based, contention-free access mechanism to reduce collisions. In Figure 9, we demonstrate the number of estimated stations (or SUs) compared to the actual number of stations in the network with the help of an error graph. We can see that the error bars of the estimated number of stations do not deviate much than the standard values for the true number of stations in the network. This verifies the accuracy of the probabilistic estimation method used in the group split algorithm. Moreover, we observe a nominal estimation noise (error) when probability parameter *a* is set to 0.85.

**Figure 10:**
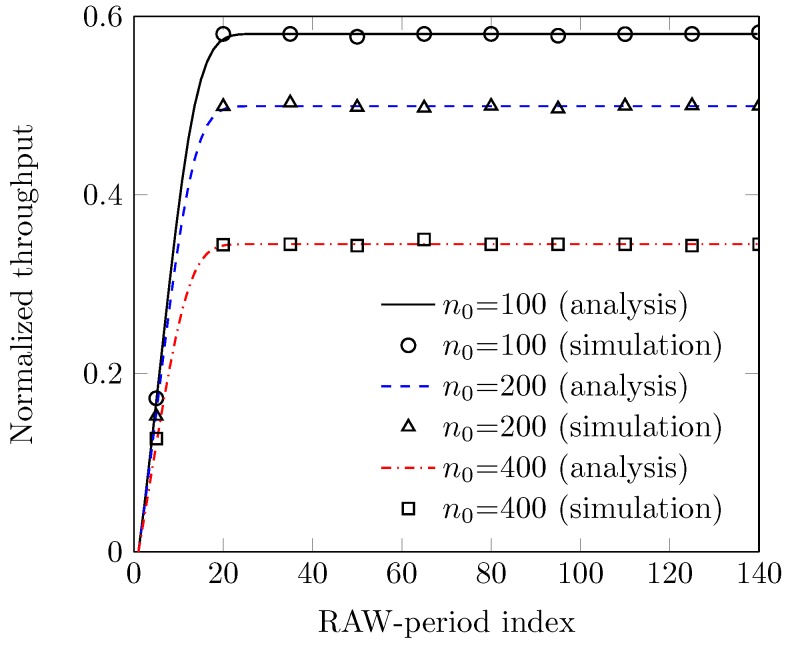
Throughput with grouping of stations (at various $n_{0}$s).

In Figure 10, we describe the normalized throughput with grouping of stations in the RACIR-rts/cts system for various sizes of the target group $n_{0}$, and the number of RAW-periods. We observe that throughput of the system initially remains low, and then monotonically increases with the increase in RAW-periods up to a certain limit, and, thereafter, it becomes steady. This is because, in early RAW-periods, the size of the groups remains larger due to less grouping of the stations and so the maximum backoff counter from $W_{0}$ becomes smaller for many SUs in each group, which results in a large number of collisions. However, the collision-resolution power of the system gradually increases as the RAW-period index goes by, because, with time, the size of the groups gets smaller and closer to that of the target group and thus, increases system performance. When the size of the groups equals (or decreases to) the target group size, the system does not allow further grouping of the stations and so, its throughput becomes steady. However, the gap in the curves of the throughput is attributed to the various sizes of the target group. We also observe that a smaller target group size performs better due to fewer collisions, and improves system performance.

**Figure 11:**
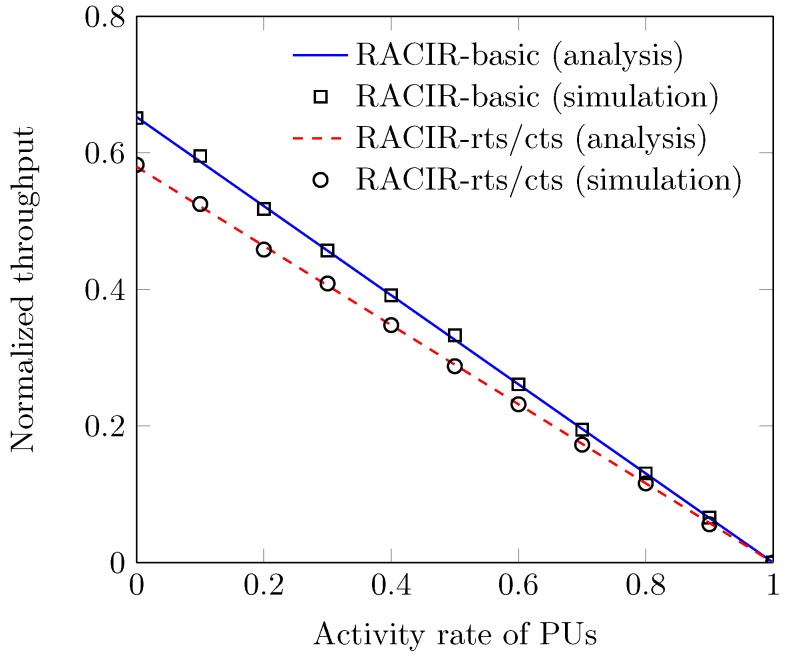
Throughput vs. activity rate of Primary Users.

**Figure 12:**
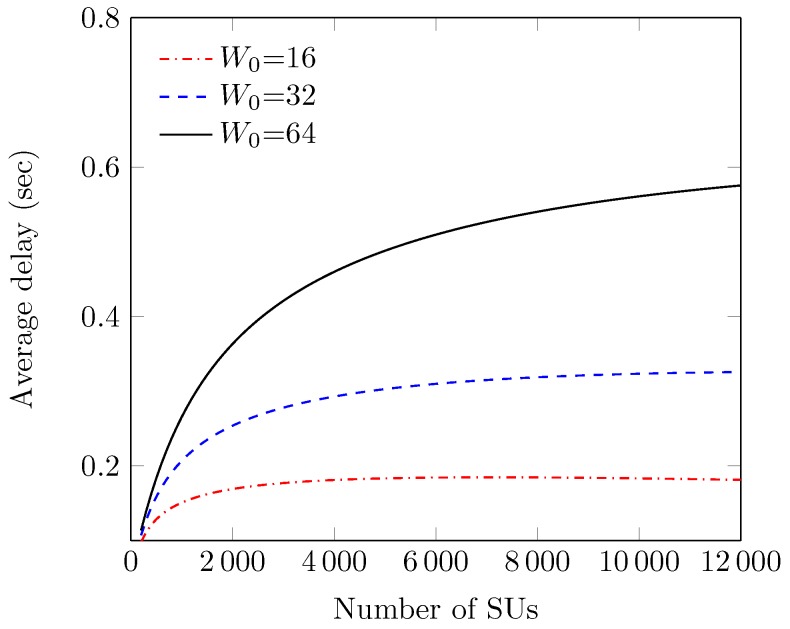
Delay vs. the number of stations (at various $W_{0}$s).

Figure 11 shows the effect of the PU activity rate $\pi_{1}$, over the normalized throughput of the RACIR protocols. We observe that values of S curves keep monotonically decreasing with an increase in $\pi_{1}$. This is because SUs increase the blocking probability with an increase in the PU detection rate in the ID slot to avoid interference, and generate more and more spectrum access to the primary network. In return, channel access to the secondary network is reduced, which ultimately decreases system throughput. We also observe that RACIR-basic achieves better performance, compared to RACIR-rts/cts, because of its direct-access mechanism. Figure 12 shows the effect of the number of SUs *N*, on average packet delay D, at different sizes of the initial contention window $W_{0}$. We observe that values of D remain higher at large values of $W_{0}$ and *N*. This is due to the fact that a large *N* can improve packet load with a higher probability of packet collisions, as calculated with Equation (9). Thus, the large $W_{0}$ yields a larger delay than that a small $W_{0}$ in order to reduce the number of collisions. Eventually, D curves (after a certain limit) reach their maximum values and, thereafter, they do not aggravate anymore, since backoff stages and buffer sizes are limited. To this end, another factor that also contributes, in part, is the grouping phenomenon due to which the scalability of the network remains under control, and so it makes the D curves insensitive to the number of SUs.

**Figure 13:**
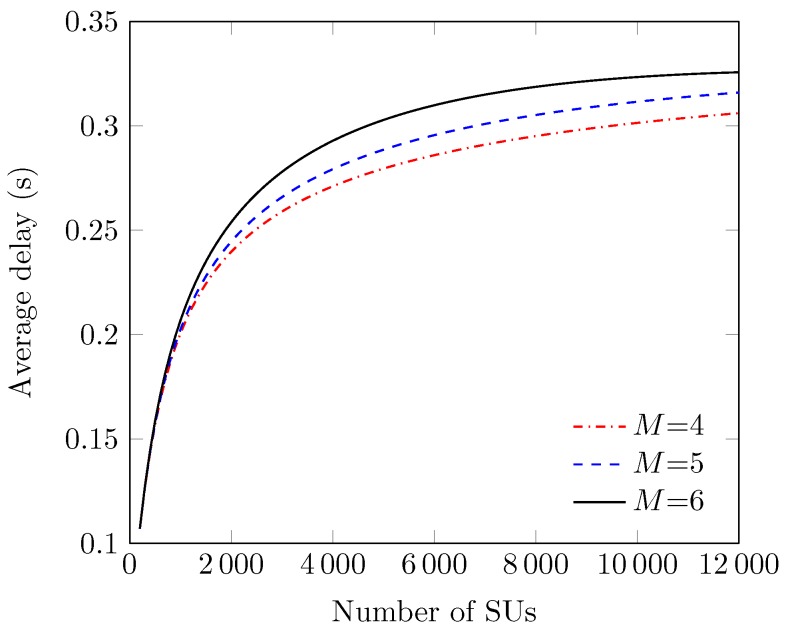
Delay vs. the number of stations (at various *M*s).

Figure 13 exhibits the effect of *N* on average packet delay D at different values of maximum backoff stage *M*. We see that values of D are an increasing function of *N*. The reason is that at a large *N*, an SU’s HOL packet has to wait longer with the increased backoff delay due to the large number of collisions. We also see that the values of D remain higher for a large *M* because it can lead to queueing the packets in order to increase the probability of successful transmission, but at the cost of increased delay.

**Figure 14:**
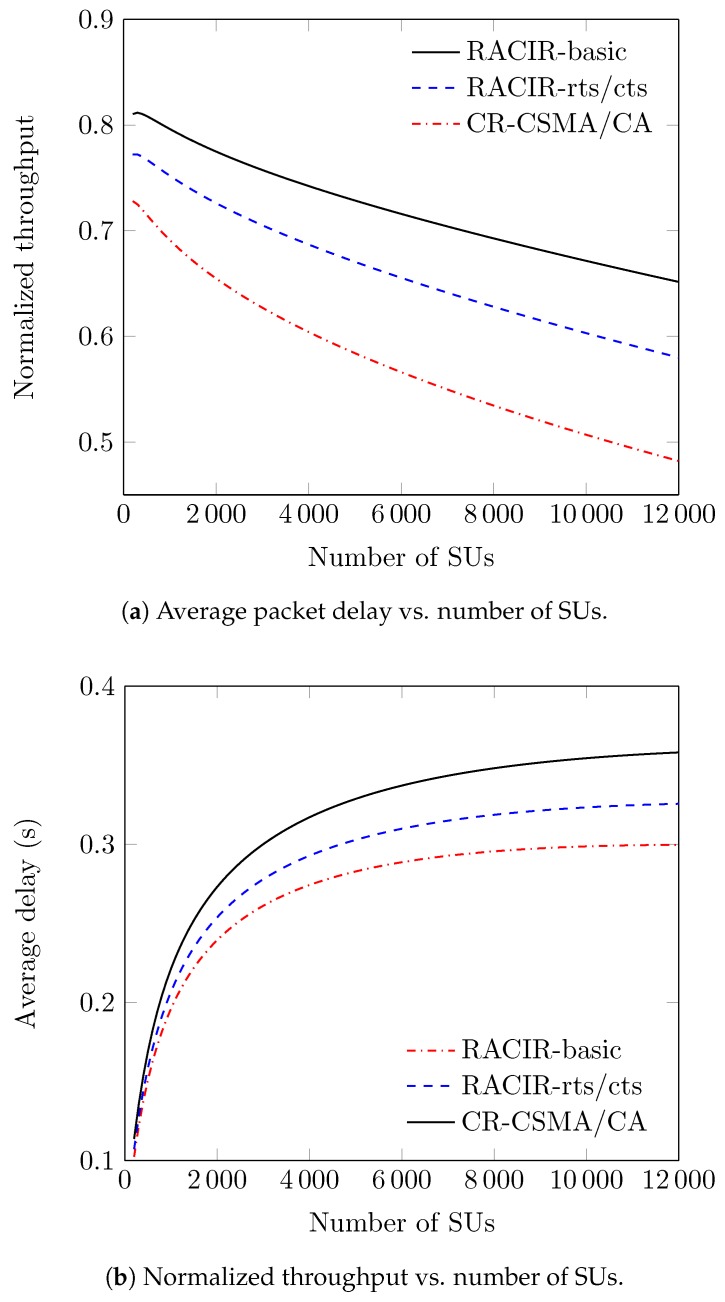

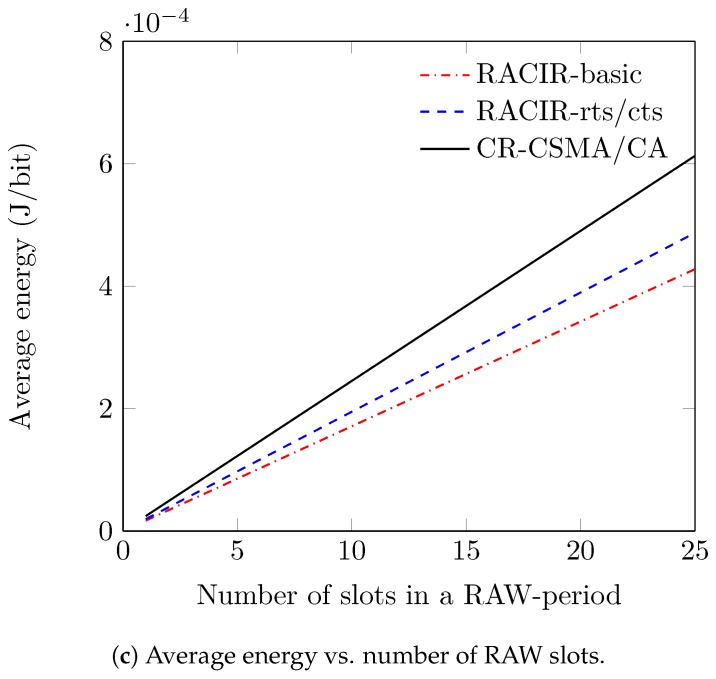
Performance comparisons between CR-CSMA/CA and RACIR protocols in a super-dense environment.

Figure 14 describes performance comparisons among CR-CSMA/CA, RACIR-basic, and RACIR-rts/cts protocols. In Figure 14a, we see that values of S decreased monotonically with an increase in *N* due in part to the following reasons. First, the maximum backoff counter chosen from $W_{0}$ becomes smaller with an increase in *N*. In due course, SUs observe frequent collisions and thus, decrease system throughput. On the other hand, SUs can be positioned into many groups with an increase in *N*. Hence, the time to rendezvous between the SUs is also increased, and that can lead to a decrease in system throughput. However, RACIR-basic outperforms RACIR-rts/cts and CR-CSMA/CA protocols due to its direct-access method. Conversely, RACIR-rts/cts outperforms the CR-CSMA/CA protocol due to its lowest possible control overhead to protect the PU receiver. Thus, it collectively spares a fraction of the bandwidth for transmission of more data packets, which ultimately maximizes system performance. We also observe that our proposed RACIR protocols have lower packet delay than CR-CSMA/CA in Figure 14 b. Similarly, this is attributed to the lower overhead required for each successful operation of the RACIR protocols, which saves channel negotiation costs and thus, decreases the average packet delay.

We demonstrate the average energy consumption per delivered bit (in Joules) for the various numbers of RAW slots in Figure 14c. We can see that energy consumption increases with the increase in the number of slots *S*, in a RAW-period. This is evident because the length of the RAW-period is a function of *S*, which ultimately increases the wakeup time of the stations, and thus increases energy consumption. However, the gap between the energy curves is attributed to the unique cost of MAC protocols. We observe that both variants of RACIR outperform CR-CSMA/CA protocol due to their smart operations that lead to save the fraction of energy in the system, which is what is critical for IoT applications. Hence, the proposed RACIR can be a good candidate MAC for CR-based IoT networks.

## 9. Conclusions

In this paper, we introduced a carrier sense Restricted Access Collision and Interference Resolutions (RACIR) protocol for CR-based IEEE 802.11ah networks. Therein, each station first estimates the number of active stations with a new group split algorithm to enable contention-free grouping in a decentralized manner in order to resolve collisions. Then, the stations in each group periodically access the channel with a contention-based random access mechanism and avoid interference with the PU receiver. In this mechanism, stations first conduct carrier sensing for the EIFS interval, and then they choose a backoff counter at random. When the backoff counter reaches 0, the transmitter first exchanges RTS and CTS packets with the receiver and then transmits data under CSMA/CA. Right after the data transmission begins, the transmitter selects an Interference Detection (ID) slot from an ID period at random, in order to conduct spectrum sensing. Similarly, the receiver conducts spectrum sensing when it receives the first bit of a data packet. Meanwhile, if either the transmitter or receiver finds a PU to be active, it broadcasts a JAM signal to stop transmission and then invokes a blocking state for a predefined period. Otherwise, both of them continue transmitting and receiving the data frames. Finally, the transmitter receives an ACK from the receiver and completes the transmission. In this way, our protocol resolves the scalability issue and avoids the hidden primary terminal problem. We investigated the RACIR performance, for both RTS/CTS access control and the basic access mechanism, in terms of throughput, delay and energy consumption. We observe that our RACIR outperforms CR-CSMA/CA, under the scenario of interest, due to less overhead. Furthermore, analytic results reveal that RACIR is suitable for super-dense IoT environments because its performance is insensitive to the size of the network.

## Figures and Tables

**Table 1:** IEEE 802.11 system timing parameters (μs).

IEEE Standard	DIFS	SIFS	Idle Slots
802.11a	34	16	9
802.11b	50	10	20
802.11n	34	16	9
802.11ac	34	16	9
802.11ah	264	160	52

**Table 2:** Summary of the symbols.

Symbol	Description
$\pi_{1,j}$	Probability of activity for SU *j*’s neighboring PUs
$\pi_{0,j}$	Probability of inactivity for SU *j*’s neighboring PUs
$\alpha_{i}$	SU *j*’s misdetection probability
$\beta_{i}$	SU *j*’s false alarm probability
$\hat{N}$	Estimated number of stations
*N*	True number of stations
*K*	Number of estimation slots
$n_{0}$	Target group size in the network
*k*	Number of non-accessed estimation slots
Ω	Probability of non-accessed estimation slots
$\hat{\Omega }$	Maximized probability of non-accessed estimation slots
κ	Probability of channel clearance from the activity of the PU
*a*	Probability parameter (0<a≤1)
S	Normalized throughput of RACIR
D	Average delay of an SU’s HOL packet
$P_{\varepsilon_{i}}$	Probability of event $\varepsilon_{i}$ (i=1,⋯,4)
$T_{\varepsilon_{i}}$	Length of time for event $\varepsilon_{i}$ (i=1,⋯,4)
$P_{n_{i}}$	Probability of SU *n*’s $\varepsilon_{i}$ slots (i=2,⋯,4)
*E*	Channel bit transmission rate
*p*	Transmission failure probability
*q*	Interference probability
$\tau_{j}$	Probability of transmission from SU *j*
$W_{0}$	Initial contention window size
*m*	Backoff stage of an SU
$W_{m}$	Contention window size at the *m*-th stage
*M*	Maximum stage in the backoff process
*H*	PHY and MAC header size
*P*	Packet payload size of an SU
σ	Duration of a backoff slot
E[R]	Average duration of a backoff slot
Ps	Probability of successful transmission
Ptr	Probability of packet transmission from SUs
*X*	Number of backoff slots an SU’s HOL packet observes
*S*	Number of slots in a RAW-period
E	Average energy per delivered bit
$P_{wr}$	Transmission/Reception power of the radios

**Table 3:** Default system configurations.

Parameter	Value
MAC header	272 bits
PHY header	120 bits
Payload size	8184 bits
RTS size	160 bits + PHY header
CTS/ACK size	112 bits + PHY header
SIFS duration	10 μs
EIFS duration	70 μs
Slot duration (σ)	20 μs
ID slot duration	50 μs
Propagation delay	1 μs
Rx/Tx-Tx/Rx switching time	20 μs
Neighboring PU activity rate ($\pi_{1}$)	0.1
Spectrum sensing duration (SS)	0.5 ms
Channel bit transmission rate (*E*)	1 Mbps
Max. backoff stages (*M*)	5
Min./Max. window size ($W_{0}$/$W_{M}$)	32/1024
Transmission/Reception power ($P_{wr}$)	100 mW
